# Canadian Rural-urban Differences in End-of-life Care Setting Transitions

**DOI:** 10.5539/gjhs.v4n5p1

**Published:** 2012-06-25

**Authors:** Donna M. Wilson, Roger Thomas, Katharina Kovacs Burns, Jessica A. Hewitt, Osei-Waree Jane, Robertson Sandra

**Affiliations:** 1 Faculty of Nursing, University of Alberta, Edmonton, Canada; 2 Faculty of Medicine, University of Calgary, Calgary, Canada; 3 Health Sciences Council, University of Alberta, Edmonton, Canada; 4 Faculty of Physical Education and Rehabilitation, University of Alberta, Edmonton, Canada

**Keywords:** rural, health services utilization, palliative, end-of-life care, transitions, mixed-methods research

## Abstract

Few studies have focused on the care setting transitions that occur in the last year of life. A three part mixed-methods study was conducted to gain an understanding of the number and implications or impact of care setting transitions in the last year of life for rural Canadians. Provincial health services utilization data, national online survey data, and local qualitative interview data were analyzed to gain general and specific information for consideration. Rural Albertans had significantly more healthcare setting transitions than urbanites in the last year of life (M=4.2 vs 3.3). Online family respondents reported 8 moves on average occurred for family members in the last year of life. These moves were most often identified (65%) on a likert-type scale as “very difficult,” with the free text information revealing these trips were often emotionally painful for themselves and physically painful for their ill family member. Eleven informants were then interviewed until data saturation, with constant-comparative data analysis conducted for a more in-depth understanding of rural transitions. Moving from place to place for needed care in the last year of life was identified as common and concerning for rural people and their families, with three data themes developing: (a) needed care in the last year of life is scattered across many places, (b) travelling is very difficult for terminally-ill persons and their caregivers, and (c) local rural services are minimal. These findings indicate planning is needed to avoid unnecessary end-of-life care setting transitions and to make needed moves for essential services in the last year of life less costly, stressful, and socially disruptive for rural people and their families.

## 1. Introduction

In most developed and developing countries, urbanization has been and continues to be a predominant trend (Cristancho, Garces, Peters & Mueller, 2008; Decker & Higginson, 2007). In Canada, less than 20% of citizens live in rural or remote areas across 95% of this large country, with many areas entirely uninhabited and others in what appears to be an inevitable process of depopulation (Statistics Canada, 2008, 2009). Urbanization is widely considered a consequence of national economies moving away from agricultural and natural resource bases, and with people shifting in response to this major industrial and social change. Although urbanization has many benefits, there is considerable concern worldwide that rural people have less access to health care (Hansen, Cartwright & Craig, 2011; Lynch, 2012; Menec, Nowicki, & St. John, 2011; Wilson et al., 2006). In Canada, where all citizens have equal rights to health care and where the healthcare system is designed for universal access as a consequence of public funding and administration, most healthcare services including palliative care specialist services are now only situated in city hospitals (Cohen et al., 2012; McCall & Johnston, 2007; Robinson et al., 2010). The impact of this consolidation or centralization of specialists and high-tech healthcare services in cities, a situation that is not confined to Canada, has been compounded by technological advancements that have made it possible for the vast majority of diagnostic tests and treatments to be provided in ambulatory venues such as out-patient or day-surgery clinics (McCall & Johnston, 2007; Neutel et al., 2005, 2006). Consequently, ill rural people not only need to travel to cities for most diagnostic, treatment, and palliative services; but also travel repeatedly to cities for health care that previously would have been provided consecutively during one inpatient hospital stay. This shift to ambulatory and urbanized health care may be particularly problematic for terminally-ill people, as health care is often necessary in the last year of life (Lynch, 2012; Wilson et al., 2009b). Some studies have found hospital deaths are more common among rural versus urban people as a result of their need to travel to cities for health care (Gruneir et al., 2007; Houttekier et al., 2010; Wilson et al., 2009b). However, dying in a distant city hospital is not considered a “good” rural death (Wilson et al., 2009a).

Although there is much concern about the frequent use of healthcare services by dying people and also by frail older people (Menec et al, 2011), most end-of-life care occurs at home, with temporary or permanent moves out of the home to obtain health and supportive care often required in the last year of life (Menec et al., 2010). Many end-of-life studies to date have focused on the transition from curative to palliation care, a shift that is often characterized as delayed and problematic (McCall & Johnston, 2007). It is now widely understood that transitions in healthcare goals and healthcare teams typically occur for most terminally-ill persons over the last year of life as death becomes increasingly inevitable (Abarshi et al., 2010; Giles et al., 2009; Marsella, 2009). Other end-of-life transition studies have focused on the loss of independence from functional or cognitive decline near death - without addressing the practical implications of moving a terminally-ill or dying person from one care setting to another (Davidson et al., 2007). In addition, although transitions or developments in healthcare techniques and venues have been researched (Neutel et al., 2005, 2006), end-of-life care setting transitions have rarely been studied. There is little insight then into the frequency and impact of moves on dying persons and their families, and particularly ruralites with their greater travel burden; a major knowledge gap as each move could involve many challenges and different types of transitions (Hansen et al., 2011). The mixed-methods study described here bridges this gap in understanding end-of-life care setting transitions by comparing the number and types of moves to obtain healthcare services in the last year of life for rural versus urban decedents in Alberta, a Canadian province, and gaining additional insight into the impacts of these moves or care setting transitions on rural persons through an online survey and qualitative interviews.

## 2. Background

Urban and rural healthcare services in Canada and elsewhere typically differ now in scope, size, and increasingly existence; for instance, many rural areas in Canada do not have a local hospital and many also lack family physicians (McDonald & Conde, 2010; Menec et al., 2010, 2011; Robinson et al., 2010; Roth & Barsi, 2005). With healthcare services essentially centralized in city hospitals and often provided out of urban ambulatory care venues, rural people with serious life-threatening illnesses could make many trips now to one or more cities for health care (Chan et al., 2006; Hart et al., 2005). These trips can be lengthy; in Canada, 50% of rural people live far away from any urban centers (Kelley, 2007; McGrath et al., 2007; Wilson et al., 2006). As transportation options in rural Canada are often limited to privately owned and operated vehicles, ill ruralites typically rely on themselves or family members and friends to transport them to and from urban healthcare facilities (Wilson et al., 2009a). These informal caregivers may need to be absent from their homes and other responsibilities for one or more days, as travelling for an out-patient or day-surgery procedure could involve a day’s journey to and another day’s journey following that procedure; a major concern for farmers, ranchers, trappers, and small business owners. Travelling long distances in Canada, especially in winter and at short notice, is costly and can be highly problematic. For instance, public funding for ambulances is only provided when a physician orders a transfer from one hospital to another. When an ambulance is called to take a person from their home to a hospital, this cost is either addressed out of pocket or through private healthcare insurance. This cost is proportional to the travel distance, with rural persons paying much more than urbanites. Travelling is thus a greater burden for ill rural people and their families or friends as compared to urbanites who have shorter distances to travel and a greater range of less expensive transportation options, including taxis and city buses or other public transportation.

General health status constitutes another major difference between rural and urban populations (Canadian Institute for Health Information [CIHI], 2006). In Canada, rural people are typically less healthy than their urban counterparts; with this difference identified as explaining their greater comparative use of inpatient hospital beds and emergency departments (CIHI, 2006; Stratton et al., 2007). Rural populations in Canada and elsewhere also tend to be older, another factor that increases their healthcare need while also raising the complexity of any care setting transitions (Ansari et al., 2006; Blackwell et al., 2009; Dumont et al., 2009; Evans et al., 2003; Lemstra et al., 2009; Orsi et al., 2010; Statistics Canada, 2010; Wilson et al., 2006; Wilson et al., 2009b). In addition, rural populations in Canada and other countries are typically less affluent than urban ones, with low income as another factor for both reduced health and higher healthcare needs (Blackwell et al., 2009).

Understanding the number, types, and impact of healthcare setting moves or transitions in the last year of life is important in Canada and elsewhere, as this information can be used to further planning for appropriate and possibly more cost-effective end-of-life care. Research evidence is also needed to avoid care setting transitions that are traumatizing to the terminally-ill individual and their family (Burge et al., 2005). Evidence on care setting transitions could thus have a significant impact on health policy, such as through helping to engender a policy to ensure basic end-of-life services exist in every rural community for local accessibility (duPlessis et al., 2001; Wilson et al., 2008).

## 3. Methods

As indicated above, a three part mixed-methods study was designed to gain information on end-of-life care setting transitions for rural persons. Further detail will be provided below on the first component, undertaken to compare the number and types of moves in relation to obtaining healthcare services in the last year of life for rural versus urban decedents in Alberta, a western Canadian province. Information on the number and impacts of moves to obtain health and supportive care in the last year of life was sought through an online national survey, followed by local qualitative interviews for a more in-depth understanding of rural care setting transitions. The University of Alberta’s Health Research Ethics Board approved this mixed-methods study.

### 3.1 Health Services Utilization Data and Analyses

To describe and compare care setting transitions in the last year of life across rural and urban Albertans, a secondary analysis of complete population-level province-wide inpatient hospital and ambulatory (emergency room, out-patient clinic, and day-surgery) care data was undertaken. Upon request, all data routinely collected over two recent years (April 1, 2005 through March 31, 2007) in inpatient hospital and ambulatory databases were provided along with registry data to the research team in electronic format. All three datasets contained individual-anonymous data, with a unique and consistent number assigned per person to enable data consolidation within and across datasets, and also from one year to another. The data were provided in 2009 by Alberta Health and Wellness to the research team after all research ethics and administrative approvals had been gained, and a data access fee was paid.

All 19 398 Albertans who died in the 2006-07 fiscal year (April 1 through March 31), with these clearly identified as such through being coded as deceased in one or more of the three datasets, were initially included in this quantitative phase of the study. Although 28.5% of these 19 398 decedents were never admitted for inpatient hospital care in the last 365 days of life, all had received one or more ambulatory healthcare services in the last year of life, and so all cases were initially retained for data analyses. All had evidently travelled out of their home or nursing home for healthcare services. However, when the registry and other data for all 19 398 decedents were more fully screened, 1 600 cases were deleted. All data for 379 decedents younger than 12 months of age at the time of death and 1 221 additional persons who had not resided in the province for a full year before death were removed, leaving 17 798 cases in the final dataset.

The total number of healthcare setting transitions for each person was then computed, with a healthcare setting transition defined as any geographic move that an individual made in the last 365 days of life for healthcare purposes. Care setting transitions typically occurred in groups of two, as individuals were often admitted to a hospital for inpatient or ambulatory care from a home or nursing home and then discharged from this hospital to their home or nursing home. Less often, three care setting transitions were grouped together, with these often in the form of a person being admitted to a local hospital, transferred to a larger hospital, and then discharged from that hospital to home. Although this computation of healthcare setting transitions that was gained through this analysis of health services utilization data is complete as healthcare services data are carefully and consistently collected in the province, one limitation of this study is that these health services data do not reflect the total number of care setting transitions that could have taken place in the last year of life. Permanent or temporary moves from one home to another home or from a home to a nursing home were not captured in these healthcare datasets.

Postal code data were used to classify each decedent as having resided in an urban or rural area. Urban areas in Canada are defined as Census Metropolitan Areas (CMAs) and Census Agglomerations (CAs), with these understood as large urban areas or adjacent fringe areas with a large degree of urban integration (Racher, Vollman & Annis, 2004). CMAs and CAs have minimum populations of 100 000. In the final collective dataset that was constructed to contain comparable aggregate but individualized data gained for each individual from the three datasets, those persons who lived within CMAs and CAs were designated as “urban;” all others were designated as “rural.” Rural/urban peoples were then examined for differences in age, total number of healthcare setting transitions, number of inpatient discharges, number of inpatient days per hospital stay, total number of healthcare procedures received in the year, total number of visits to large provincial, medium-sized regional, and/or small rural hospitals; and total length of all inpatient hospital stays.

Logistic regression analyses were then conducted to seek differences between 13 859 ruralites and urbanites (a smaller subject set, as these persons alone had complete data on all variables of interest) on the number of care setting transitions, number of inpatient discharges, number of procedures, and number of visits to provincial, regional and/or rural hospitals. The dichotomous variable for the logistic regression was rural versus urban with the other variables entered through the step-wise method as covariates. Age as a categorical variable (age 65 or older-0 and younger than 65-1) was entered first into the model. The number of care setting transitions was entered next. The third set of covariates included the number of inpatient discharges and number of procedures. In the final block, the number of visits to the various hospitals was entered. All data were analyzed using the SPSS (Version 18) computer software program.

### 3.2 Online Data Collection and Analyses

Around the same time that the provincial healthcare data were requested and the team were waiting for it to be supplied, a simple survey was devised by a multi-disciplinary Canadian team and posted online to seek cross-Canada information on the total number of moves that ruralites made to receive healthcare and supportive care services in the last year of life, the place or places of this care, and the impact of moving from one care setting to another. The questionnaire was uploaded to a website in 2009, after local pilot testing to ensure both few and clear questions, as well as ease of opening and completing this online survey. Information about this survey and a link to it were then circulated to over 50 Canadian palliative care and rural organizations for distribution to their members. This survey was posted for six months, with instructions indicating that respondents should be those who could provide information about a rural Canadian who had died in the last year. As such, the respondent could be an urbanite, but they were to report on a rural decedent. This survey was also aimed at gathering information about people who had died in the last year, with this participation requirement included in the information letter and on the survey tool to reduce recall or memory issues and gain current information. As many health system and healthcare changes occur every year, information older than one year was viewed as unlikely to reflect current healthcare and end-of-life realities. A total of 108 respondents provided partial or full data in the six month interval before the survey was taken down from the website. A descriptive summary of data obtained from these 108 respondents was then completed using the SPSS computer program.

### 3.3 Qualitative Data Collection and Analyses

As indicated, qualitative interviewing was also undertaken to gain an understanding of the implications and practical impacts of care setting transitions in the last year of life for rural persons. All interviews took place in Alberta, the home province of the researchers. Alberta was considered appropriate for this study because of its vast rural areas and highly centralized healthcare services, with specialist healthcare services (including palliative care specialist services) situated in only a few cities. Qualitative interviews were chosen as the most appropriate method for gathering in-depth insight into the implications and practical impacts of care setting transitions that occur in the last year of life. To minimize the possible burdens of research on terminally-ill persons and their families, a decision was made to interview only bereaved family members. Participation was voluntary, and potential informants were asked to call an Edmonton-area telephone number they would have either read on notices about the study placed in prominent rural newspapers, community service settings such as hospitals and medical clinics, or rural grocery store billboards, and/or would have heard about through friends or family members. Potential participants were screened by telephone to ensure all criteria for the study were met. Individuals had to be willing and able to talk openly about the death of a loved one and state that they were able to have this discussion without it causing them undue stress or distress. Participants were also told they could withdraw from the study at any time. None withdrew, probably because the screening process eliminated persons who became emotional when discussing the study. The family member’s death had to have happened in the past year, so that current healthcare service and other information would be obtained rather than information about historical issues or contexts. Although each informant did not need to reside in a rural area, the person who died had to be identified as a rural resident. A rural definition was not provided as it is most important that the decedent was considered to have been a “rural” resident (du Plessis et al., 2001; Racher et al., 2004; Wilson et al., 2009a).

Throughout 2009, key informant English-speaking family members of rural Albertan decedents were interviewed about the implications and practical impacts of moves made in the last year of life to obtain needed end-of-life services. After signing a consent form, one taped 50-75 minute semi-structured interview was conducted with each participant. Field notes were taken prior to, during, and after each interview to capture additional information of relevance that was seen, heard, or otherwise gained by the researcher. This researcher was experienced in qualitative data gathering and analysis. Although the study did not strictly conform to grounded theory methodologies, in keeping with what are now relatively standard grounded theory data gathering and analysis methods, ongoing constant-comparative data analysis using the transcribed interview and fieldnotes data occurred until data saturation (Chiovitti & Piran, 2003). Data saturation is the point when no new data are gained and no new meanings from existing data emerge (Guest et al., 2006). Saturation was determined to have been reached after 11 persons had been interviewed. Data analysis consisted of coding key findings, categorization or grouping findings, and thematic development as the study progressed (Chiovitti & Piran, 2003). Two researchers acting independently of each other coded and categorized the data soon after each interview, with discussion quickly following to rectify differences, and to determine which additional questions or probes needed to be asked in subsequent interviews. The two researchers worked together to group categories into themes. This data gathering and analysis approach was used as one month or more elapsed between interviews, a time gap that was largely due to the scarcity of rural decedents and the need for family members to hear about the study and then be willing and able to talk about end-of-life care setting transitions. A theory was not developed, as the researchers felt additional research involving different ethnic or other groups in additional rural areas was needed for a robust theory to be developed.

## 4. Results

### 4.1 Healthcare Services Utilization Findings

The mean age of the 17 798 decedents was 72.1 years (*SD=19.8*), with 73.2% aged 65 or older and 52.2% males. The total healthcare setting transitions for all decedents over the year prior to their death date averaged 3.5 (range=1-41, median=3, *SD=3)*.The mean number of inpatient hospital separations was 1.6 (*SD=1.7*), with 41 inpatient hospital days of care accumulated on average over the year (*SD=48.6*, Range=0-396). Also on average, 2.8 emergency room, 5.0 out-patient, and 2.5 day-surgery visits per decedent occurred in the last year of life. The average length of each inpatient stay was highest in the large provincial hospitals (*M*=37.7, *SD*=48.2), followed by small rural hospitals (*M*=32.5, *SD*=39.4) and then mid-sized regional hospitals (*M*=27.6, *SD*=32.8). Only 47% of these deaths took place in hospital, however. One third of all decedents (*N=5,317*) were designated as having received specialized palliative care in the last year of life, although 85% of these persons had only one palliative-related admission or palliative care service before death.

As shown in Table 1, 18.2% of the decedents were ruralites, with 70.0% of ruralites and 74.0% of urbanites 65 years of age or older. More ruralites (55.4%) than urbanites (51.5%) were male. Ruralites had significantly more healthcare setting transitions than urbanites (M=4.2 vs 3.3, t=13.0, df=13857, *p*< .05). Ruralites also had more (i.e. 2.6) procedures on average performed in the last year of life as compared to urbanites (2.4), another significant difference (t=3.1, df =13857, *p*< .05). Significant rural-urban differences were also manifested in inpatient hospital discharges (t=10.5, df =17796, *p*< .05), with 2.0 discharges on average for ruralites versus 1.5 for urbanites.

**Table 1:** Rural versus urban socio-demographic and healthcare service utilization differences

Variable	Total 17,798(100%)	Rural 3,239(18.2%)	Urban 14,559(81.8%)	*p*
**Gender (N)**				
Male	9288(52.2%)	1794(55.4%)	7494(51.5%)	.00
Female	8510(47.8%)	1445(44.6%)	7065(48.5%)	
**Age group (N)**				
Younger than 65	4761(26.8%)	973(30%)	3788(26%)	.00
Older than 65	13037(73.2%)	2266(70%)	10771(74%)	
**Care setting transitions**	3.5	4.2	3.4	.00
**Inpatient discharges**	1.6	2.0	1.5	.00
**Total stay (days)**	41.0	40.8	41.1	.79
**Number of procedures**	2.4	2.6	2.4	.00
**Visits to facilities**				
Provincial Hospitals	1.3	.72	1.38	.00
Regional Hospitals	0.3	.33	.27	.00
Rural Hospitals	0.7	1.65	.61	.00
**Length of inpatient stay (days)**				
Provincial Hospitals	37.7	28.6	38.8	.00
Regional Hospitals	27.6	22.9	35.1	.00
Rural Hospitals	32.5	32.8	32.4	.75

Mean differences were assessed with independent-samples t-tests or chi square tests

Ruralites accessed small hospitals more often that urbanites (M=1.65 for ruralites, M=0.61 for urbanites), and urbanites more often accessed large provincial hospitals than ruralites (M=0.72 for ruralites, M=1.38 for urbanites). Urbanites had significantly longer stays in provincial hospitals (M=38.8 for urbanites versus M=28.6 for ruralites, t=6.5, df=9461, *p*< .05), as well as regional hospitals (M=35.1 for urbanites versus M=22.9 for ruralites, t=3.8, df=2180, *p*< .05). No rural/urban differences in average length of stays for small rural facilities were found (M=32.8 for ruralites, M=32.4 for urbanites). Ruralites and urbanites also did not differ significantly in the total number of days spent in hospital in the last year of life, as ruralites averaged 40.8 days and urbanites averaged 41.0 days (t=0.3, df=13857, *p*> .05).

### 4.2 Logistic Regression Analyses

When age was entered into the model, a significant difference between ruralites and urbanites was noted (χ^2^ (1)=8.3, *p*< .05). If the individual was rural, the odds of being older increased by .67. The model continued to remain significant with the addition of healthcare setting transitions (χ^2^ (2)=153.9, p<.05). The greater the number of transitions, the odds of the individual being a rural decedent increased by 1.15. Similarly, model fit increased with the addition of total inpatient discharges, as well as number of procedures (χ^2^ (4)=235.1, *p*<.05). The -2 log likelihood decreased from Model 2 to Model 3 with 4 variables, from 12785.1 to 12703.9, further demonstrating that these variables explained differences between ruralites and urbanites. The addition of number of visits to provincial, regional, and rural hospitals in the final model did not change this significance (χ^2^ (7)=1587.9, *p*< .05). The significance of the full model with all variables implies that it is a better fit for understanding rural-urban differences than the constant-only model. Summary statistics for the final model are presented in Table 2.

**Table 2:** Summary (final model) of logistic regression for rural and urban decedents (N=13 859)

Predictors	B	SE B	OR	CI	Wald	*P*
Age group	- .41	.06	.67	.60- .74	54.13	.000
Caresetting transitions	.14	.03	1.15	1.08-1.23	16.87	.000
Inpatient discharges	- .68	.06	.51	.45- .57	125.46	.000
Number of procedures	- .04	.01	.96	.95- .98	27.29	.000
Provincial facility visits	.89	.03	2.45	2.31-2.60	951.23	.000
Regional facility visits	.44	.03	1.55	1.46-1.64	200.54	.000
Constant	1.76	.05				

*Note*: OR indicates Odds Ratios. CI indicates 95% confidence intervals. The significance of the regression coefficients were tested by the Wald’s chi-square statistic

### 4.3 Online Survey Findings

The 108 respondents to the online survey reported a total of 1 to 39 moves in the last year of life, with an average of 8, the vast majority of which were from home to hospital and then from hospital to home. Five were from one home to another home, and only one was from a home to a nursing home. Travelling was reported as being needed to access services in many different cities and/or towns, and also in multiple places within one city. For instance, one person reported “overall, he went to 3 different hospitals in one city, and for other health care at 4 more cities/towns. Most of the travelling was for his monthly check up and if he was OK, then another trip was for his monthly chemotherapy. But in the end, he had to go in for radiation every day for 2 weeks. We did a lot of travelling together.” Discontinuity of care issues were also identified, such as by the comments “we would get there and find they had forgotten to order his chemotherapy, so we had to go home and come back the next day” and “we never knew who we would meet, as the doctors were all new to us, and they usually did not know much about her.”

These care setting transitions were most often (65%) identified as “very difficult” on a 5-point likert scale. The free text information from all those who reported very difficult transitions revealing these trips were emotionally painful for themselves and physically painful for their ill family member. For instance, one respondent reported “it was terrible taking him in as we did not want to hear the diagnosis” and another reported “he could barely walk as he was in such pain, car trips hurt him, and when we got there, we had to walk around a really big hospital.” In addition, these trips were often reported as stressful as they were done at all times of the year, and this travelling was costly. Lost wages and having to pay for hotels, meals, parking, and hotels were listed as issues. The comments provided revealed considerable stress and anxiety over these trips.

### 4.3 Qualitative Interview Findings

The 11 key informants were all adults and most (10/11) were female. These persons reported on their spouse (2/11) or a parent (9/11) and although standard socio-demographic data were not collected on these persons and the decedents, it became evident that most were aged 65 or older and all older persons were retired. Most (8/11) of the decedents were not married as they neared the end of life. The data gained through the 11 key informants revealed three themes: (a) needed care in the last year of life is scattered across many places, (b) travelling is very difficult for terminally-ill persons and their caregivers, and (c) local rural services are minimal. These themes are described and illustrated below.

#### 4.3.1 Theme One: Needed Care in the Last Year of Life Is Scattered Across Many Places

In the last year of life, travelling to many different places was required. According to these participants, there were 8 to 39 care setting transitions per person, although one participant said “we were on the road so much, I can only remember and count 39, but it was probably more. He was so very ill after each chemotherapy treatment that I would need to take him back to a hospital for dehydration treatment. He was usually kept in for a few days. He would get home and recover a bit before we would need to go in and get checked out for the next monthly treatment. I got to dread those treatments. This cycle started after tests and more tests to get an accurate diagnosis, every one of them was in a different hospital or clinic. If we had only known what was involved, and that it was all futile in the end…” Both the participant and her partner who died of cancer were working at the time of his diagnosis, and she continued to work full-time off their farm throughout the year over which his health deteriorated. He quit working six months before his death, and although the majority of days in the last year of his life were spent at home, he died in hospital. This hospital was not their local hospital, which was a 15 minute drive away from their farm, but instead a regional hospital that was in the same city as a member of his immediate family (a 45 minute drive from the farm). He did not wish to die in the large cancer hospital where he had received treatments nor in the two large city hospitals where he had been admitted for dehydration. Those hospitals involved more than 60 minutes of travel time for his wife and other family members.

All other participants also outlined that travelling to many different places was required for prescribed tests and treatments, and for palliative or end-of-life services as it became evident that death was inevitable and drawing near. Illustrative quotes included: “we had to go to many different hospitals, doctor’s offices, and clinics to get the care that he needed; sometimes it was in the big city four hours drive and then the awful drive in rush hour there, or to other cities; we were going in every direction from our farm” and “she had her doctor in town, and her cancer doctor was in the city where she got her chemotherapy, but she had tests in three other cities. I don’t know what was worse, her cancer or having to get to so many different clinics and hospitals on time for her doctor’s visits. We were always worried we would get there late or not at all. The worst was when she had to see a physiotherapist as she had trouble walking, and we got lost on the way. She cried and cried that day…. It was awful.”

Not only were many different cities visited but often many different places in each city. For instance, one participant said that their family got to know the big city well, as “we went to all four big hospitals, each one in a different part of the city, and we also went to about five other places where he needed to be seen or examined. We also travelled in for supplies and medications, as our local pharmacy and drug store did not have what he needed (when he was at home). They would be able to get it in over a few days, but we usually needed it sooner than that. We used to joke that we could run a taxi service in the city, as we got to know it so well.” Another participant indicated that their family could not understand why their father’s cardiologist worked out of one hospital and had an office nearby, but tests were done when he experienced heart failure in two different hospitals and four other ambulatory clinics. She said “we realized quickly we were the glue. We held it all together.” Travelling was thus revealed as being a common aspect of life for terminally-ill rural persons and their families as the care required is not consolidated in one place.

#### 4.3.2 Theme Two: Travelling is Very Difficult for Terminally-Ill Persons and Their Family Caregivers

Travelling was revealed as very difficult for both the terminally-ill person and their family, and for many different reasons. Illustrative quotes include: “we dreaded travelling, we knew the news would be bad, and we never knew if they would be ready for us; we would get there, and wait and wait,” “each trip was long and tiring, and I was afraid to drive in bad weather and with him, he was so sick,” “he could not work and I had to take time off work; it was a good thing we lived close enough to the city so we could get in and out in one day (and avoid the added expense of hotels and restaurant meals),” “he couldn’t drive anymore when he got sick, so I had to drive and he was very critical of my driving, but he was upset and angry with everything then, I don’t blame him as he was so sick. He would cry out in pain when I hit a bump, and I would cry then too,” and “we were kept really busy that last year, with all the trips and all. I wonder if it would have been better to have had more time together as a family at home, without that driving. We lost touch with friends too. I guess you can’t change what happened, but he was dying and that time was precious.”

Another set of quotes illustrates how difficult travelling was for the terminally-ill person and their family: “I would drive him in and park in front of the hospital doors. He had early morning appointments and I hardly slept the night before. I would help him out and he would slowly go to the admitting desk while I parked the car. He could barely walk and he would not use a wheelchair. I always worried he would fall, and I knew it hurt him a lot to walk. I could park at the meters close by if I had enough change, or park underground but that was really expensive. It was better (to park underground) though as I never knew how long he would be. When I got to the admitting desk, he was either there looking very frustrated because it took so long to get attention or gone off somewhere. He could be anywhere in the hospital, and I had to find him as he needed me to be there;” and “I did most of the driving for his tests and treatments. One friend was able to take time off work and drive him once in and out of the city, and one niece was able to do that. That really helped, as there was no bus or handy van to get him in and out of the city. Some towns have handy vans but ours is too small. We were just lucky that he had one friend who was not too old to drive and a niece who was unemployed for a while, we kind of lost touch with everyone else.” Although some rural people may not travel much in the last year of life, all of the 11 participants indicated travelling was common and this need to travel presented many difficulties. Travelling was complex and costly; with social, emotional and physical impacts as well.

#### 4.3.3 Theme Three: Local Rural Services Are Minimal

The participants revealed that local-area services of all kinds were minimal, with some entirely missing and others difficult to access. Three quotes illustrate this theme: “we have a local hospital, but it does not have much to offer; we had to go in to the city for tests and treatments,” “there isn’t a doctor in the town anymore and the closest hospital is an hour away. That was bad, but it was worse as none of her children or close relatives lived in the area anymore,” and “she lived in the senior’s lodge in town after Dad died, and when she got sick she was taken to a small hospital close by but ended up in a large city hospital. When it was clear Mom was dying, I brought her to my house (on the farm) as the lodge would not take her back (as lodges do not provide care to ill persons). She died before I could get any help from our local community homecare agency and church group.” With minimal local rural services, travelling to cities to access needed services was required.

## 5. Discussion

This mixed-methods study revealed rural decedents in a province had undertaken significantly more healthcare setting transitions in the last year of life as compared to urban decedents, including nearly 50% more trips for inpatient hospital care. Although the actual difference in average formal healthcare setting transitions between ruralites and urbanites was relatively small (4.2 vs 3.3), it is important to remember that rural and urban people tend to have vastly different circumstances impacting their access to and use of healthcare services (Barbera et al., 2010; Hansen et al., 2011; Roth & Barsi, 2010), even in a country such as Canada with universal access because of publicly-funded health care. The online survey and interviews helped to reveal these circumstances, while highlighting the issue that healthcare setting transitions in the last year of life can and do often involve difficult travelling and related burdens for the dying ruralite and their family. Future research should be done to determine if these burdens are shared by dying urbanites and their families, or if there are differences.

This additional research is recommended, as any trips taken could be for scheduled tests and treatments, but many could be needed on short notice as terminally-ill people are prone to sudden changes in their health and thus their healthcare needs. Much uncertainty is present with terminal illnesses; as it is possible for the person to die quickly, linger indefinitely in relatively stable or changeable health, or experience rapidly and significantly deteriorating health. As such, terminally-ill people and their family caregivers need to be prepared for multiple healthcare setting transitions, ones that can involve short or perhaps extended hospital stays (Casey et al., 2005; Virnig et al., 2006). Rural people, and low income rural people in particular, may need assistance with travelling, particularly as low income is a commonly identified factor for unmet healthcare needs in Canada and other countries (Peterson & Gitaker, 2010). In addition, as previous rural research has shown the importance of having family members who can transport ill persons so they access healthcare services (Arcury et al., 2005), ruralites without families or without local family members are likely to need assistance with accessing or obtaining healthcare services.

The quantitative data analysis phase of this study also revealed healthcare setting transitions more often involved ambulatory or same-day healthcare services as compared to inpatient hospital care. Although much concern has been raised about the high use of hospitals by terminally-ill and dying people (Menec et al., 2010), the findings of this study emphasizes that most end-of-life care in the last year of life occurs at home. Families are clearly a very important component of end-of-life care (Hansen et al., 2011). The finding that less than half of all decedents died in hospital and that 28.5% of all decedents in the province were never admitted to an inpatient hospital bed in the last year of life help to emphasize the possibility that a person can become ill, have a series of diagnostic tests that establish a terminal illness, see one or more specialists, receive a range of curative and possibly palliative treatments, and die without spending a single night admitted to a hospital bed for “professional” care. This observation is a more likely fit for urbanites that ruralites in Alberta, however, as rural decedents were more often hospitalized in the last year of life, despite rural and urban persons having roughly the same average number of total hospital days accumulated over this year.

The finding of a greater average number of inpatient hospital stays for ruralites over urbanites (M=2.0 vs M=1.5 respectively) is a particularly important one, as it raises many considerations. It is commonly believed that distance to hospital is negatively correlated with hospital admissions, with urban people then having an advantage for accessing hospitals because of their closer proximity to them (Salinas et al., 2010). However, a lack of palliative care and other specialists in rural communities would necessitate travel to city hospitals and ambulatory care venues (Menec et al., 2011). In the province of Alberta, it is also possible that terminally-ill rural Albertans are more often admitted to hospital because of the provincial government’s decision during the mid-1990s recession to keep small rural hospitals open while downsizing large city hospitals. From 1993 to 1995, one half of all 13 000 hospital beds were closed (mostly in large cities) in Alberta, at the same time that a nearby province (Saskatchewan) was instead closing 52 of its smallest (rural) hospitals to similarly reduce healthcare expenditures. Most of the small rural hospitals in Alberta have remained open since then, although specialists and advanced or high-tech health services are now centralized in large provincial city hospitals and to a lesser extent mid-sized regional hospitals. Rural Albertans can therefore access one or more small local hospitals, in addition to mid-sized regional hospitals and large provincial hospitals; while urbanites would normally only access their own city’s (regional or provincial) hospitals. Albertans can choose to go to any hospital emergency department in the province and they may be sent home or admitted to that hospital or transferred to a hospital that has the services that they need. As outpatients, they may request a specific facility to have a booked test or procedure in, but normally they are told which facility to attend since their specialist works there or it has the type of equipment and expertise required. In Alberta, there are 10 large provincial hospitals and 10 mid-sized regional hospitals which provide a wide range of services, as compared to over 80 small rural hospitals which typically have a much smaller range of services (i.e. few will have surgical services capacity). In short, the greater number of accessible rural and other hospitals to ruralites may explain the higher number of healthcare setting transitions and hospital admissions for rural decedents. The higher average number of procedures performed on ruralites in the last year of life may reflect their more frequent trips to hospitals, although this finding could also reflect a lower state of health among rural persons as was indicated in a previous national study (CIHI, 2006). This higher number could also indicate a problem of repetitious or futile healthcare services.

Other factors for the higher number of rural healthcare setting transitions, such as a lack of palliative care and other specialists in rural communities (Robinson et al., 2010), may also necessitate travel to urban centers after local-area generalist healthcare services have been accessed. Regardless, it is important to raise the issue that despite this study’s finding of the higher number of healthcare setting transitions for rural persons, ruralites may not access all needed end-of-life care services because of transportation or other issues. There are many practical problems associated with transporting terminally-ill persons from one place to another (Casey et al., 2005; Roth and Barsi, 2008; Virnig et al., 2006). McDonald and Conde’s (2010) study finding that older ruralites made fewer visits to general practitioners and medical specialists as compared to older urbanites was explained by those researchers through the simple fact that rural people had to travel longer distances and with fewer transportation options.

The online and qualitative data also showed care setting transitions for rural people in the last year of life are highly concerning. Given the fact that this was the last year of life, and these trips were problematic, it is important to consider if all trips taken were essential. Although travelling in the last year of life may be needed to definitively diagnose a terminal illness and for symptom management, it would be prudent for healthcare professionals and policy makers to seek to eliminate unnecessary trips for repetitive and/or futile care. The higher rate of procedures performed on rural versus urban persons in the last year of life suggests rural people are at particular risk of repeated and/or futile care.

Reducing the negative impacts of care setting transitions is another area for attention. Not only should there be efforts to reduce the emotional, physical, and other strains on terminally-ill individuals of having to move outside of their homes and communities to obtain healthcare services; but efforts should also focus on reducing the family’s caregiver burden associated with moving their ill family member from one place to another. Rural municipalities could recognize and address this issue such as by having local multi-purpose vans that can be used to transport ill persons and their accompanying family member(s) in and out of the local community for needed healthcare services. Rural municipalities could also advocate for travelling clinics, such as a weekly or monthly palliative care in-community clinic (Robinson et al., 2010).

## 6. Conclusion

Care setting transitions in the last year of life are often needed but are concerning for many reasons. The mixed-methods study reported here compared rural and urban healthcare setting transitions over the last year of life and sought additional insights about these transitions for rural persons. Some important differences in rural versus urban end-of-life healthcare services use were identified. Ruralites had a greater number of care setting transitions, inpatient hospitalizations, and procedures performed. Care setting transitions were then identified through the online survey and qualitative interviews as challenging for dying rural people and their families. Although much more research is needed to continue to improve end-of-life care in both rural and urban areas, policy and practice should focus on specific issues to facilitate needed care setting transitions for rural peoples, while also eliminating unnecessary moves.
